# Visceral fat area as a predictor for macrovascular complications in patients with type 2 diabetes mellitus

**DOI:** 10.3389/fendo.2026.1636998

**Published:** 2026-02-12

**Authors:** Mengli Sun, Wenting Chen, Zhenzhen Lin, Enling Ye, Mengmeng Peng, Liangmiao Chen, Hong Yang

**Affiliations:** Department of Endocrinology, The Third Affiliated Hospital of Wenzhou Medical University, Wenzhou, Zhejiang, China

**Keywords:** brachial-ankle pulse wave velocity, carotid intima-media thickness, predictive model, type 2 diabetes, visceral fat area

## Abstract

**Objective:**

This study aimed to investigate the association between visceral fat area (VFA) and markers of atherosclerosis in patients with type 2 diabetes mellitus (T2DM), with or without macrovascular complications (MVC). Additionally, the study sought to determine the optimal VFA threshold for predicting MVC in individuals with T2DM.

**Methods:**

This retrospective study included 1, 176 patients with T2DM and 289 healthy individuals enrolled between August 2018 and May 2022. Participants were classified into three groups: healthy controls, T2DM without MVC, and T2DM with MVC. Demographic characteristics, clinical parameters, and laboratory data were collected. VFA, carotid intima-media thickness (CIMT), and brachial-ankle pulse wave velocity (baPWV) were measured. Univariate analysis was conducted for variable selection, followed by multivariate logistic regression to identify independent predictors. A risk prediction model was constructed. Model calibration was assessed using the Hosmer–Lemeshow goodness-of-fit test, and predictive performance was evaluated using the area under the receiver operating characteristic curve (AUC).

**Results:**

Baseline comparisons across the three groups revealed a progressive increase in VFA from healthy controls to individuals with T2DM alone and to those with T2DM and MVC (*P* < 0.001). Subgroup analysis within the T2DM group showed significant differences in VFA between the CIMT(-)baPWV(+) and CIMT(-)baPWV(-) subgroups, as well as between the CIMT(+)baPWV(+) and CIMT(-)baPWV(-) subgroups. Multivariate logistic regression identified age, systolic blood pressure, weight, body mass index, VFA, and triglycerides as independent predictors of MVC in T2DM (all *P* < 0.05). The predictive model was defined as: Logit(P) = –11.942 + 0.083 × age + 0.035 × systolic blood pressure - 0.030 × weight - 0.109 × BMI + 0.054 × VFA + 0.154 × triglycerides. The model achieved an AUC of 0.867, with a sensitivity of 72% and a specificity of 86%.

**Conclusions:**

VFA is an independent predictor of MVC in patients with T2DM, demonstrating superior predictive value compared to traditional indicators such as BMI. The predictive model developed in this study shows high accuracy, supporting early identification of high-risk individuals and enabling implementation of personalized interventions.

## Introduction

Type 2 diabetes mellitus (T2DM) is a metabolic disorder characterized by insulin resistance and hyperglycemia, with a steadily increasing global prevalence. As of 2024, the total number of adult individuals with diabetes was approximately 589 million worldwide, including 148 million in China. This figure is projected to rise to 168.3 million by 2050 (1). Among Chinese adults aged 20-79, diabetes-related mortality accounts for up to 10.6% of all-cause deaths (1). Compared with individuals without diabetes, those with T2DM have a significantly higher risk of developing atherosclerotic cardiovascular disease (ASCVD), including a 72% increased risk of heart attack and a 52% increased risk of stroke (1). ASCVD remains the leading cause of mortality among individuals with diabetes.

Due to its often asymptomatic progression and potential for sudden onset, early identification of individuals at elevated risk for ASCVD is critical for timely diagnosis and intervention. Carotid intima-media thickness (CIMT) is widely recognized as an early marker of atherosclerosis (2). It is assessed using B-mode ultrasound, offering a non-invasive and well-validated method for detecting early arterial structural changes (3). Additionally, brachial–ankle pulse wave velocity (baPWV) serves as another non-invasive parameter for evaluating arterial stiffness (4, 5).

Obesity is one of the most significant risk factors for the development of T2DM and is closely associated with its occurrence (6). Among Chinese adults with diabetes, more than 60% are overweight or obese (7). Body mass index (BMI) is the most commonly used metric for assessing obesity and overweight globally. However, BMI cannot adequately reflect fat distribution. While obesity is a known risk factor for various cardiovascular diseases, the coexistence of obesity and T2DM further increases the risk of macrovascular complications (MVC) (8). Nevertheless, two large meta-analyses have reported that overweight or obese individuals with diabetes, particularly elderly patients, may have a better prognosis than those with normal BMI, a phenomenon referred to as the “obesity paradox” (9, 10). Notably, these studies used BMI as the sole criterion for defining obesity. However, increasing evidence has suggested that abdominal obesity, rather than general obesity, is closely associated with cardiovascular risk (11–13).

Compared with other ethnic groups, East Asians tend to have relatively mild overall obesity but a greater tendency toward visceral fat accumulation, making abdominal obesity more prevalent (14). A survey of Chinese adults with T2DM found that 39.7% had abdominal obesity (15). In diagnosing metabolic syndrome, waist circumference has largely replaced BMI as a measure of abdominal obesity. However, waist circumference alone cannot distinguish whether abdominal obesity stems from visceral or subcutaneous fat accumulation. Visceral fat area (VFA) is a gold standard for diagnosing abdominal obesity, which offers an accurate and direct measure of visceral fat accumulation. A whole-body magnetic resonance angiography study has shown that increased visceral fat volume is associated with early signs of atherosclerosis (16). Another study on untreated obese T2DM patients without cardiovascular risk factors found that visceral, but not subcutaneous, fat was related to early atherosclerosis (17). However, other studies have reported that, after adjusting for traditional cardiovascular risk factors, the association between visceral fat and atherosclerosis disappeared (18–21).

In this study, we aimed to explore the relationship between VFA and two atherosclerosis markers, CIMT and baPWV, in T2DM patients with or without MVC. Furthermore, we investigated the optimal threshold value of VFA for predicting MVC in patients with T2DM.

## Methods

### Participants

This retrospective study was conducted among patients with T2DM who were admitted to the Standardized Metabolic Disease Management Center of our hospital between August 2018 and May 2022. All enrolled patients met the World Health Organization (WHO) 2017 diagnostic criteria for T2DM.The exclusion criteria were: (1) diagnosis of a diabetes type other than T2DM; (2) presence of severe hepatic or renal dysfunction; (3) Acute infections, recent surgeries, trauma, or malignancies; (4) known endocrine disorders such as hypothyroidism, polycystic ovary syndrome (PCOS), or Cushing’s syndrome, which substantially affect body fat distribution;and (5) incomplete clinical or biochemical data. A total of 1, 176 patients with T2DM were included in the final analysis.Additionally, 289 healthy individuals who underwent routine physical examinations at the hospital during the same period were selected as the control group. Inclusion criteria for healthy participants were: (1) Fasting blood glucose <5.6mmol/L; (2) absence of malignant tumors, tuberculosis, or other chronic debilitating diseases; (3) no history of acute infections, traumatic injuries, or acute cardiovascular, hepatic, gastrointestinal, renal, or cerebrovascular conditions;(4) no history or current evidence of hypothyroidism, Cushing’s syndrome, polycystic ovary syndrome, or other conditions that may cause abnormal visceral fat distribution; and (5) no personal or family history of diabetes.All participants underwent standardized assessments, including questionnaire surveys, physical examinations, laboratory tests, and measurements of VFA, CIMT, and baPWV.

The study protocol was conducted per the Declaration of Helsinki and approved by the Ethics Committee of Ruian People’s Hospital(NO.YSZM2021059). Written informed consent was obtained from all participants.

### Data collection

A standardized questionnaire was used to collect baseline information, including sex, age, height, weight, waist circumference, hip circumference, smoking status, past medical history (e.g., hypertension, hyperlipidemia), and duration of diabetes. Smoking history was defined as continuous or cumulative smoking for six months or longer at any point in life (22). BMI was calculated as weight in kilograms divided by the square of height in meters (kg/m^2^). BMI categories were defined as follows: normal weight (18.5 ≤ BMI < 24.0), overweight (24.0 ≤ BMI < 28.0), and obesity (BMI ≥ 28.0) (23). The waist-to-hip ratio (W/H) was calculated by dividing waist circumference by hip circumference. All participants fasted for 8–12 h before peripheral venous blood samples were collected the following morning. Laboratory results of triglycerides (TG), total cholesterol (TC), low-density lipoprotein cholesterol (LDL-C), high-density lipoprotein cholesterol (HDL-C), fasting blood glucose (FBG), and glycosylated hemoglobin (HbA1c) were obtained. All biochemical parameters were measured using the same laboratory protocols for both healthy participants and patients with T2DM.

VFA and subcutaneous fat area (SFA) were assessed using a dual bioelectrical impedance analysis (BIA) instrument (HDS 2000; Omron Co., Ltd., Kyoto, Japan) (24). The assessment procedure was as follows: (1) participants were instructed to fast beginning at 20:00 on the evening prior to the examination; (2) on the day of the measurement, participants lay in a supine position with wrists, ankles, and abdomen exposed; hand and foot electrode clips, along with an abdominal electrode belt, were applied; (3) after a deep inhalation and breath-hold, VFA and SFA measurements were recorded. A VFA ≥ 100 cm^2^ was defined as indicative of increased visceral adiposity (25).

CIMT and baPWV were used to evaluate arterial sclerosis. All assessments were conducted with subjects in a supine position, the neck fully exposed, and the head tilted slightly backward and away from the sonographer to minimize muscular tension due to hyperextension. CIMT was measured using a Doppler color ultrasound diagnostic system (Philips, Royal Netherlands) with a probe frequency of 7.5–10MHz. The intima-media thickness of the far wall of the bilateral common carotid arteries was measured approximately 1cm proximal to the carotid bifurcation. Measurements from both the left and right sides were recorded, and the greater value was used for subsequent analysis. All CIMT assessments were performed by experienced sonographers. Carotid intima-media thickening was defined as CIMT >0.9mm in either or both carotid arteries (26).

BaPWV was measured using a pulse waveform analyzer (BP-203RPE II, Form PWV/ABI, Omron Co., Ltd.). Participants remained supine for at least 10 min prior to measurement, with both upper and lower limbs positioned at heart level.Measurements were obtained for both the left and right sides, and the mean baPWV value was calculated. A baPWV value of >1400 cm/s indicated arterial stiffness (4, 5). Based on CIMT and baPWV results, patients with T2DM were further categorized into a T2DM without MCV group and a T2DM with MCV group.

### Statistical analysis

All statistical analyses were performed using SPSS 25.0 software. Continuous variables with a normal distribution were expressed as mean ± standard deviation, whereas those not following a normal distribution were presented as median and interquartile range (P25, P75). For normally distributed data, the means of multiple groups were compared using one-way analysis of variance, and pairwise comparisons were performed using the least significant difference test. For non-normally distributed data, the Kruskal-Wallis H test was applied. Categorical data were expressed as frequencies or percentages, and group comparisons were conducted using the Chi-square test. Univariate analysis was used to screen variables. Significant variables were then included in a multivariate logistic regression model to identify independent predictors and construct a predictive model for MVC in T2DM patients. The Hosmer-Lemeshow goodness-of-fit test was used to evaluate model calibration. The area under the receiver operating characteristic (ROC) curve (AUC) was used to evaluate the predictive efficacy of the model. A P-value below 0.05 was considered statistically significant.

## Results

### Baseline characteristics of participants

A total of 289 healthy individuals and 1, 176 patients with T2DM were enrolled in this study. The baseline characteristics of the participants are summarized in **Table 1**. VFA showed a progressive increase from healthy controls to patients with T2DM without complications and to those with T2DM and MVC. The median VFA values for these three groups were 55 (39.5, 75.0), 77 (62.2, 96.0), and 98 (83.0, 119.3)cm^2^, respectively. The differences in VFA among the three groups were statistically significant (*P* < 0.001) and remained significant across all pairwise comparisons (*P* < 0.001).

**Table 1:** Baseline characteristics of all participants.

	Healthy controls (n=289)	T2DM without MVC (n=386)	T2DM with MVC (n=790)	*P*-value
Gender (Male/Female)	119/170	293/93	590/200#	<0.001
Age (years)	39 (31, 52)	48 (39, 54)	56 (50, 62)	<0.001
Course of disease (years)	–	7 (3, 12)	11 (6, 14)	<0.001
DBP (mmHg)	71.24 ± 9.43	74.07 ± 9.73	77.69 ± 10.95	<0.001
SBP (mmHg)	118.40 ± 13.29	123.75 ± 15.98	138.28 ± 18.75	<0.001
Weight (kg)	59.34 ± 9.85	67.66 ± 10.59	69.27 ± 9.72	<0.05
BMI (kg/m^2^)	22.35 ± 2.65	24.56 ± 2.95	25.65 ± 2.67	<0.001
Hip circumference (cm)	76.96 ± 8.89	87.02 ± 8.0	91.57 ± 7.57	<0.001
W/H ratio	0.83 (0.78, 0.89)	0.922 (0.89, 0.958)	0.97 (0.926, 1.0)	<0.001
VFA (cm^2^)	55 (39.5, 75.0)	77 (62.2, 96.0)	98 (83.0, 119.3)	<0.001
SFA (cm^2^)	140 (104.0, 169.5)	156.2 (123.3, 189.0)	178.1 (149.8, 211.9)	<0.001
V/S ratio	0.43 (0.35, 0.53)	0.50 (0.43, 0.57)	0.57 (0.49, 0.65)	<0.001
Hypertension (%)	9.0%	21.2%	53.5%	<0.001
Hyperlipidemia (%)	36.0%	54.4%	63.9%	<0.001
Smoking (%)	18.3%	51.6%	53.2%#	<0.001
Alcohol consumption history (%)	14.9%	60.1%	63.2%#	<0.001
FBG (mmol/L)	4.8 (4.49, 5.09)	7.63 (6.49, 9.68)#	7.55 (6.27, 9.44)#	<0.001
Fasting insulin (pmol/L)	33.98 (26.78, 41.5)	56.01 (35.84, 87.53)#	56.99 (37.90, 94.86)#	<0.001
Fasting C-peptide (pmol/L)	456 (374.5, 569)	408.4 (267, 613.5)#	473.75 (315, 644)#	<0.05
HbA1c (%)	5.4 (5.1, 5.59)	9.16 (7.43, 11.79)	8.53 (7.32, 10.2)	<0.001
ALT (U/L)	18 (13.5, 26.0)	23.5 (16.0, 37.0)#	23.5 (17.0, 36.0)#	<0.001
AST (U/L)	19 (17.0, 24.0)	19 (15.0, 25.0)	19 (16.0, 25.0)	0.354
ALP (U/L)	66 (55, 80)	72 (59, 89)#	72 (59, 87.3)#	<0.001
γ-GT (U/L)	19 (14, 32)	27 (18, 45)	33 (21, 51.25)	<0.001
ALB (g/L)	44.1 (42.3, 45.7)	41.3 (39.0, 44.2)#	41.2 (38.8, 44.0)#	<0.001
BUN (mmol/L)	4.92 (4.09, 5.63)	5.05 (4.16, 5.98)	5.46 (4.60, 6.43)#*	<0.001
Cr (μmol/L)	59 (51.0, 70.0)	68 (60.0, 74.0)#	68 (61.0, 76.0)#	<0.001
UA (μmol/L)	295 (241, 356)	318 (263.5, 380.2)#	328 (279, 394)#	<0.001
TG (mmol/L)	1.02 (0.77, 1.51)	1.44 (0.98, 2.09)	1.7 (1.19, 2.42)	<0.001
TC (mmol/L)	4.78 (4.19, 5.49)	4.58 (3.90, 5.31)#	4.57 (3.88, 5.43)#	<0.05
HDL-C (mmol/L)	1.35 (1.12, 1.56)	1.0 (0.83, 1.19)#	1.01 (0.87, 1.19)#	<0.001
LDL-C (mmol/L)	2.89 (2.43, 3.58)	2.85 (2.24, 3.49)	2.77 (2.1, 3.41)#	0.003
TSH (mIU/L)	1.61 (1.17, 2.08)	1.35 (0.96, 1.92)#	1.42 (1.03, 1.99)#	<0.05
CIMT (mm)	0.55 (0.43, 0.65)	0.70 (0.60, 0.80)	0.95 (0.70, 1, 1)	<0.001
BaPWV (cm/s)	1157 (1055.3, 1283.8)	1265 (1166.4, 1334.6)	1661 (1536.8, 1861.1)	<0.001

^#^*P* < 0.05, compared with the HC group; *P < 0.05, compared with the T2DM without MVC group.

DBP, diastolic blood pressure; SBP, systolic blood pressure; BMI, body mass index; W/H ratio, waist-to-hip ratio; VFA, visceral fat area; SFA, subcutaneous fat area; V/S ratio, visceral fat area-to-subcutaneous fat area ratio; FBG, fasting blood glucose; HbA1c, glycosylated hemoglobin; ALT, alanine aminotransferase; AST, aspartate aminotransferase; ALP, alkaline phosphatase; γ-GT, γ-glutaminyl transferase; ALB, albumin; BUN, blood urea nitrogen; Cr, creatinine; UA, uric acid; TG, triglycerides; TC, total cholesterol; LDL-C, low-density lipoprotein cholesterol; HDL-C, high-density lipoprotein cholesterol; TSH, thyroid stimulating hormone; CIMT, carotid intima-media thickness; BaPWV, brachial-ankle pulse wave velocity.

### Characteristics of T2DM patients in different subgroups

Patients with T2DM were stratified into three subgroups based on CIMT and baPWV results: CIMT(-)baPWV(-), CIMT(-)baPWV(+), and CIMT(+)baPWV(+). As shown in **Table 2**, significant differences in VFA were observed between the CIMT(-)baPWV(+) and CIMT(-)baPWV(-) groups, as well as between the CIMT(+)baPWV(+) and CIMT(-)baPWV(-) groups. However, no significant difference in VFA was found between the CIMT(-)baPWV(+) and CIMT(+)baPWV(+) groups.

**Table 2:** Characteristics of T2DM patients stratified by CIMT and baPWV.

	CIMT(-)baPWV(-) (n=386)	CIMT(-)baPWV(+) (n=389)	CIMT(+)baPWV(+) (n=401)	*P*-value
Gender (Male/Female)	293/93	261/128	329/72	<0.001
Age (years)	46.50 ± 10.62	54.33 ± 9.34	57.09 ± 8.25	<0.001
Course of disease (years)	7.0 (3, 12)	10.0 (5, 14)	11.0 (6, 15)	<0.001
DBP (mmHg)	74.07 ± 9.73	78.23 ± 10.96#	77.17 ± 10.94#	<0.001
SBP (mmHg)	123.75 ± 15.98	137.92 ± 18.17#	138.63 ± 19.32#	<0.001
Weight (kg)	67.66 ± 10.60	68.72 ± 9.88	69.81 ± 9.55#	0.011
BMI (kg/m^2^)	24.56 ± 2.95	25.65 ± 2.69#	25.65 ± 2.65#	<0.001
Hip circumference (cm)	87.02 ± 8.0	91.34 ± 7.76#	91.79 ± 7.38#	<0.001
W/H ratio	0.92 ± 0.06	0.96 ± 0.06	0.97 ± 0.06	<0.001
VFA (cm^2^)	79.04 ± 23.2	103.22 ± 26.06#	101.54 ± 26.63#	<0.001
SFA (cm^2^)	159.59 ± 48.08	184.84 ± 45.71#	179.39 ± 42.68#	<0.001
V/S ratio	0.51 ± 0.11	0.57 ± 0.12#	0.58 ± 0.12#	<0.001
Hypertension (%)	21.2%	48.1%	58.9%	<0.001
Hyperlipidemia (%)	54.4%	66.1%#	61.8%#	0.003
Smoking (%)	51.6%	49.4%	56.9%	0.094
Alcohol consumption history (%)	60.1%	61.4%	64.8%	0.367
FBG (mmol/L)	7.63 (6.49, 9.68)	8.01 (6.38, 9.45)	7.43 (6.15, 9.45)	0.374
Fasting insulin (pmol/L)	56.01 (35.84, 87.53)	53.9 (37.47, 90.59)	58.50 (38.3, 102.2)	0.075
Fasting C-peptide (pmol/L)	408.4 (267.0, 613.5)	484.8 (329.5, 656.5)#	467.0 (297.5, 626.0)#	<0.05
HbA1c (%)	9.16 (7.43, 11.79)	8.56 (7.38, 10.01)#	8.52 (7.30, 10.38)#	<0.001
ALT (U/L)	23.5 (16.0, 37.0)	24.0 (16.0, 37.0)	23.0 (17.0, 35.0)	0.762
AST (U/L)	19 (15.0, 25.0)	20 (15.0, 26.0)	19 (16.0, 25.0)	0.413
ALP (U/L)	72 (59.0, 89.0)	72 (61.0, 88.0)	71 (58.0, 87.0)	0.634
γ-GT (U/L)	27 (18.0, 45.0)	34 (20.0, 55.5)#	31 (22.0, 48.0)#	<0.05
ALB (g/L)	41.3 (39.0, 44.2)	41.6 (38.8, 44.7)	41.0 (38.7, 43.4)	0.077
BUN (mmol/L)	5.05 (4.16, 5.98)	5.36 (4.46, 6.21)#	5.6 (4.67, 6.62)#	<0.001
Cr (μmol/L)	68 (60.0, 74.0)	66 (60, 74.5)	70 (62.0, 77.0)#*	<0.05
UA (μmol/L)	318 (263.5, 380.3)	323 (278.5, 385.5)	334 (279.0, 398.5)	0.091
TG (mmol/L)	1.44 (0.98, 2.09)	1.74 (1.22, 2.61)#	1.64 (1.17, 2.32)#	<0.001
TC (mmol/L)	5.58 (3.90, 5.31)	4.51 (3.82, 5.45)	4.62 (3.89, 5.35)	0.778
HDL-C (mmol/L)	1.0 (0.83, 1.19)	1.04 (0.89, 1.21)	0.99 (0.85, 1.17)*	0.014
LDL-C (mmol/L)	2.85 (2.24, 3.50)	2.65 (2.01, 3.36)	2.83 (2.16, 3.47)	0.059
TSH (mIU/L)	1.35 (0.959, 1.923)	1.44 (1.047, 1.982)	1.39 (1.01, 2.01)	0.249
CIMT (mm)	0.70 (0.6, 0.8)	0.70 (0.6, 0.8)	1.1 (1.0, 1.2)#*	<0.001
BaPWV (cm/s)	1265 (1166.38, 1334.63)	1645.5 (1525.75, 1854.75)#	1683 (1542.0, 1868.0)#	<0.001

*#P* < 0.05, compared with the CIMT(-)baPWV(-) group; *P < 0.05, compared with the CIMT(-)baPWV(+) group.

DBP, diastolic blood pressure; SBP, systolic blood pressure; BMI, body mass index; W/H ratio, waist-to-hip ratio; VFA, visceral fat area; SFA, subcutaneous fat area; V/S ratio, visceral fat area-to-subcutaneous fat area ratio; FBG, fasting blood glucose; HbA1c, glycosylated hemoglobin; ALT, alanine aminotransferase; AST, aspartate aminotransferase; ALP, alkaline phosphatase; γ-GT, γ-glutaminyl transferase; ALB, albumin; BUN, blood urea nitrogen; Cr, creatinine; UA, uric acid; TG, triglycerides; TC, total cholesterol; LDL-C, low-density lipoprotein cholesterol; HDL-C, high-density lipoprotein cholesterol; TSH, thyroid stimulating hormone; CIMT, carotid intima-media thickness; BaPWV, brachial-ankle pulse wave velocity.

### Multivariate logistic regression analysis and predictive model construction

**Figure 1:**
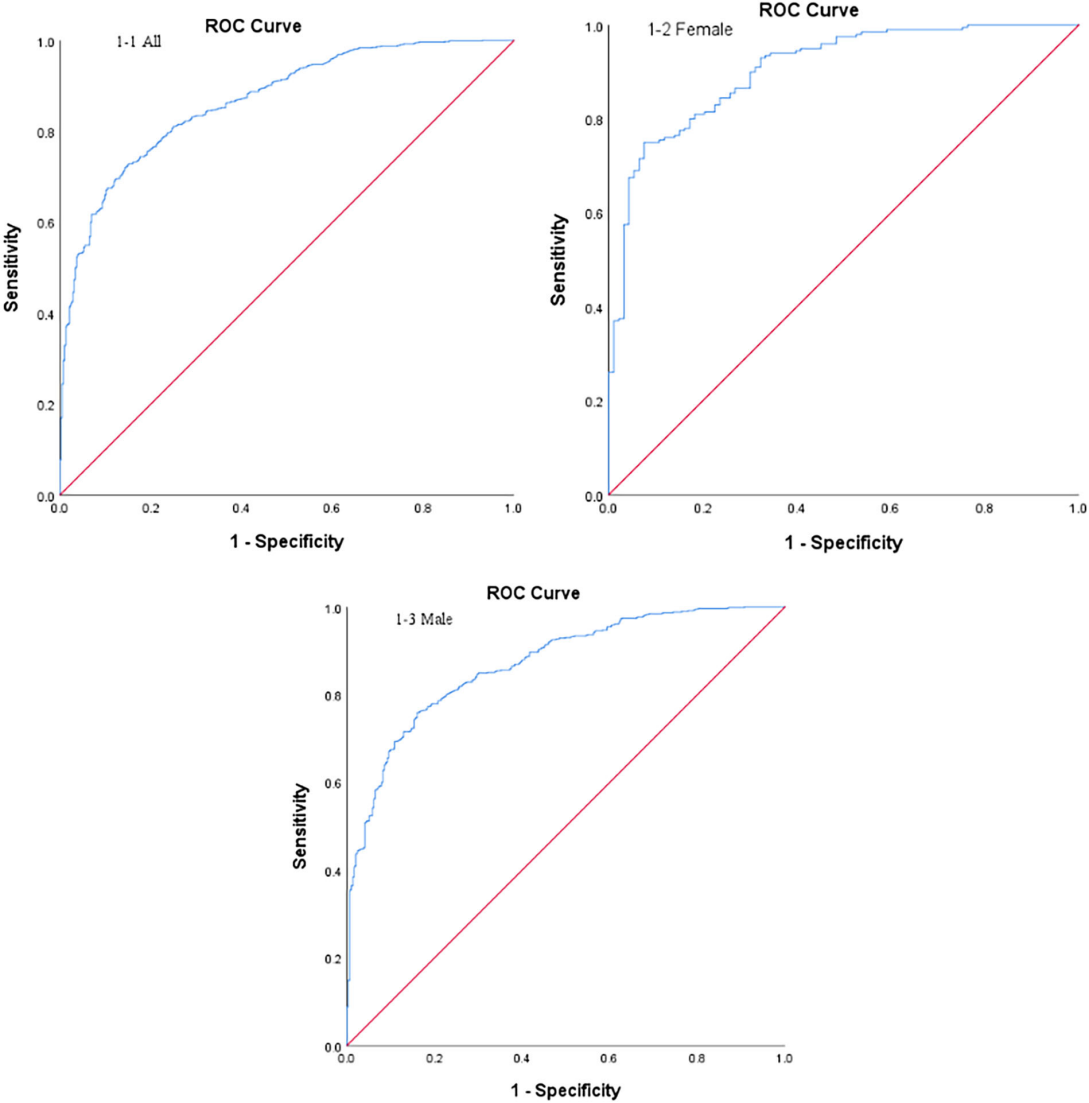
ROC curves for predicting MVC in T2DM.

Independent variables included age, systolic blood pressure (SBP), diastolic blood pressure (DBP), weight, BMI, waist circumference, waist-to-hip ratio, VFA, SFA, visceral-to-subcutaneous ratio, disease duration, history of hypertension, history of hyperlipidemia, fasting C-peptide, HbA1c, blood urea nitrogen, creatinine, and TG. The dependent variable was the absence (coded as 1) or presence (coded as 2) of MVC. Multivariate analysis showed that age, SBP, weight, BMI, VFA, and TG were independent risk factors for MVC in T2DM (*P* < 0.05) (**Table 3-1**). Based on these variables, a predictive model was developed: Logit(P) = –11.942 + 0.083 × age + 0.035 × SBP - 0.030 × weight - 0.109 × BMI + 0.054 × VFA + 0.154 × TG. This model demonstrated strong predictive performance with an AUC of 0.867 [95% confidence interval (CI): 0.847-0.888, *P* < 0.001], a sensitivity of 72%, a specificity of 86%, and an optimal cut-off value of 0.74 (**Figure 1-1**). The Hosmer-Lemeshow goodness-of-fit test indicated a good fit for the model (χ^2^ = 9.322, *P* = 0.316).

**Table 3-1:** Multivariate analysis of independent predictors for MVC in T2DM.

Variable	β	OR	95% CI	Wald Chi-square value	*P*-value
Age	0.083	1.086	1.067-1.106	78.991	<0.001
SBP	0.035	1.036	1.026-1.045	52.211	<0.001
Weight	–0.030	0.970	0.945-0.997	4.832	0.028
BMI	–0.109	0.897	0.811-0.992	4.508	0.034
VFA	0.054	1.055	1.044-1.066	98.379	<0.001
TG	0.154	1.167	1.038-1.312	6.648	0.01

**Table 3–2:** Multivariate analysis of independent predictors for MVC in the female subgroup.

Variable	Female				
	β	OR	95% CI	Wald Chi-square value	*P*-value
Age	0.092	1.096	1.053-1.141	20.268	<0.001
SBP	0.038	1.039	1.017-1.062	11.899	0.001
BMI	–0.382	0.683	0.563-0.828	15.042	<0.001
VFA	0.111	1.117	1.082-1.154	45.062	<0.001
HDL-C	–1.54	0.214	0.066-0.694	6.608	0.01
LDL-C	0.494	1.639	1.161-2.314	7.878	0.005

**Table 3–3:** Multivariate analysis of independent predictors for MVC in the male subgroup.

Variable	Male					
	β	OR	95% CI		Wald Chi-square value	*P*-value
Age	0.088	1.092	1.067-1.118	54.460		<0.001
SBP	0.035	1.036	1.025-1.048	39.574		<0.001
Weight	–0.043	0.958	0.932-0.984	9.859		0.002
VFA	0.048	1.049	1.038-1.060	74.661		<0.001
Disease duration	0.037	1.038	1.003-1.074	3.217		0.034
TG	0.376	1.456	1.187-1.787	12.943		<0.001

SBP, systolic blood pressure; BMI, body mass index; VFA, visceral fat area; TG, triglycerides; LDL-C, low-density lipoprotein cholesterol; HDL-C, high-density lipoprotein cholesterol.

**Figure 2:**
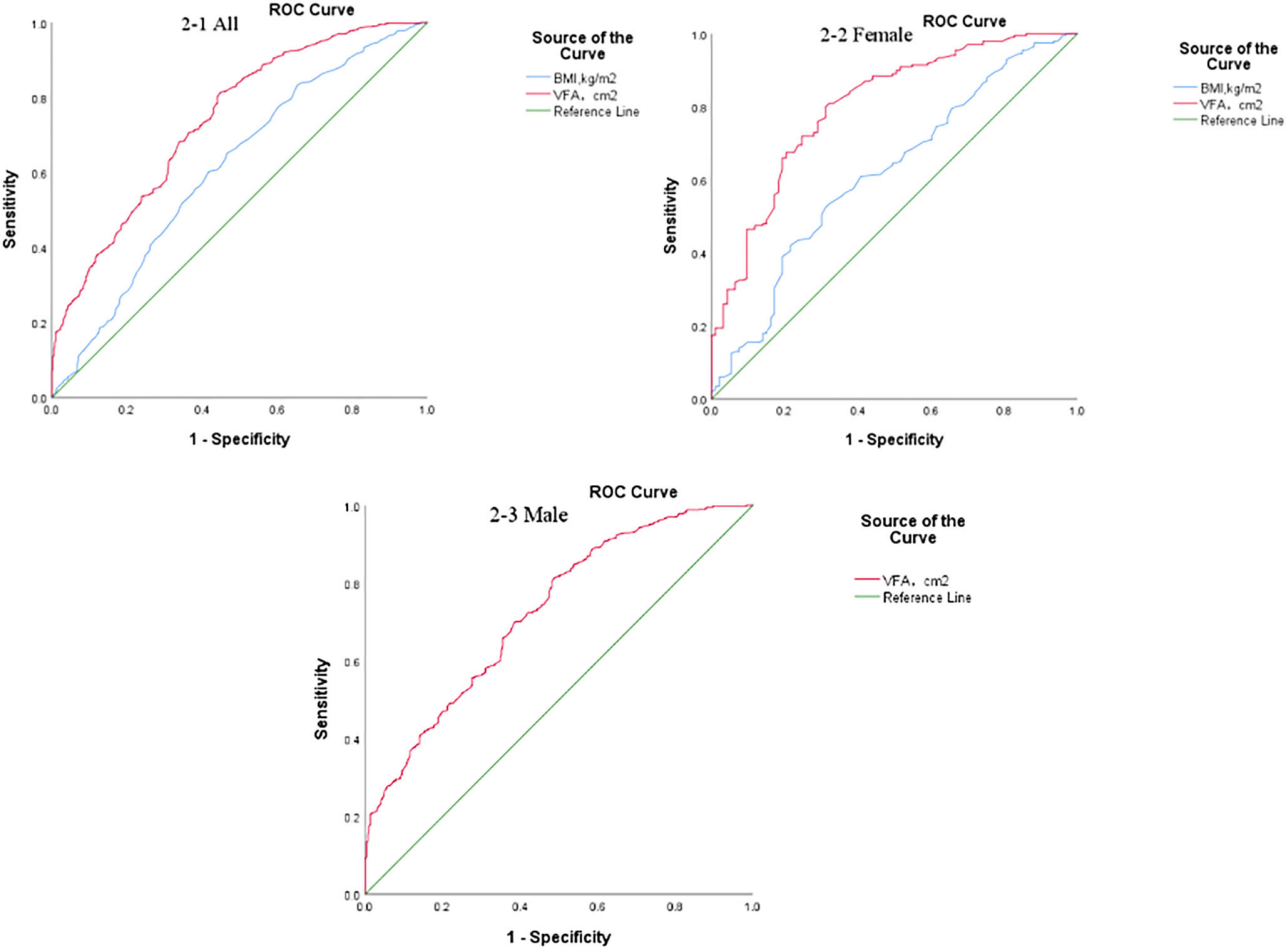
ROC curves of VFA and BMI for predicting MVC in T2DM.

Separate multivariate logistic regression analyses were conducted for male and female subgroups to identify independent predictors in different sexes. In the female subgroup, independent predictors included age, SBP, BMI, VFA, HDL-C, and LDL-C (*P* < 0.05, **Table 3-2**). The logistic regression equation was Logit(P) = –8.644 + 0.092 × age + 0.038 × SBP - 0.382 × BMI + 0.111 × VFA - 1.54 × HDL-C + 0.494 × LDL-C. This model yielded an AUC of 0.905 (95% CI: 0.870-0.941, *P* < 0.001), with a sensitivity of 75%, a specificity of 92.5%, and a cut-off value of 0.77 (**Figure 1-2**). Model calibration was acceptable (χ^2^ = 6.562, *P* = 0.584). In the male subgroup, age, SBP, weight, VFA, disease duration, and TG were identified as independent predictors (*P* < 0.05, **Table 3-3**). The regression model was Logit(P) = –10.17 + 0.088 × age + 0.035 × SBP - 0.043 × weight + 0.048 × VFA + 0.037 × disease duration + 0.376 × TG. The AUC for this model was 0.870 (95% CI: 0.847-0.894, *P* < 0.001), with a sensitivity of 75.9%, a specificity of 84.0%, and a cut-off of 0.69 (**Figure 1-3**). The Hosmer-Lemeshow goodness-of-fit test suggested that the model fit well (χ^2^ = 13.762, *P* = 0.088).

### Comparison of the predictive value of VFA and BMI for MVC in T2DM

In the overall study population, the AUC of VFA for predicting MVC in T2DM was 0.740 (95% CI: 0.710-0.770, *P* < 0.001), with a cut-off value of 80.05 cm^2^, a sensitivity of 80.3%, and a specificity of 56.0%. The AUC of BMI was 0.612 (95% CI: 0.577-0.647, *P* < 0.001), with a cut-off value of 24.45 kg/m^2^, a sensitivity of 65.2%, and a specificity of 53.4%. The AUC of VFA was significantly higher than that of BMI (Z = 5.44, *P* < 0.001), indicating that VFA had superior predictive performance (**Figure 2-1**).

In female patients, VFA also demonstrated stronger predictive ability. The AUC of VFA was 0.80 (95% CI: 0.747-0.856, *P* < 0.001), with the same cut-off value of 80.05 cm^2^, a sensitivity of 80.0%, and a specificity of 68.8%. The AUC of BMI was 0.618 (95% CI: 0.549-0.688, *P* = 0.001), with a cut-off value of 25.45 kg/m^2^, a sensitivity of 52.5%, and a specificity of 68.8%. The differences in AUC were statistically significant (Z = 4.04, *P* < 0.001), confirming the superior predictive value of VFA (**Figure 2-2**).

Among male participants, the AUC of VFA for predicting MVC in T2DM was 0.726 (95% CI: 0.691-0.761, *P* < 0.001), with a cut-off value of 79.3 cm^2^, a sensitivity of 81.5%, and a specificity of 51.2% (**Figure 2-3**).

## Discussion

This retrospective, cross-sectional study demonstrated a strong association between VFA and the presence of MVC in patients with T2DM. A progressive increase in VFA was observed across study groups—from healthy control subjects to patients with T2DM and further to those with T2DM complicated by MVC, which is consistent with findings from previous studies (27–30).

Obesity, a chronic metabolic disorder characterized by excessive fat accumulation, increases the risk of early cardiovascular complications in individuals with diabetes (31). However, adipose tissue distribution varies significantly among individuals, particularly in the relative proportions of visceral and subcutaneous fat (32). Emerging evidence has highlighted the critical role of visceral fat accumulation in the pathogenesis of both diabetes and MVC (33–35). Several mechanisms may explain this association:(1) Obesity constitutes a chronic inflammatory state in which visceral adipose tissue secretes pro-inflammatory cytokines such as interleukin-6 and tumor necrosis factor-α, contributing to vascular endothelial dysfunction (36);(2) Enhanced lipolytic activity in visceral fat leads to elevated circulating free fatty acids, which are transported via the portal vein and exacerbate insulin resistance, thereby indirectly promoting atherosclerosis (37);(3) Visceral fat accumulation is frequently associated with dyslipidemia—characterized by elevated TG, increased LDL-C, and reduced HDL-C—all of which contribute to vascular injury (38).

For the first time, this study systematically investigated the changes in VFA and structural (CIMT) and functional (baPWV) markers of arteriosclerosis in the diabetic population. Our findings revealed that the VFA in the CIMT(-)baPWV(+) group was significantly higher than that in the CIMT(-)baPWV(-) group, suggesting that elevated VFA may be linked to early vascular dysfunction, as reflected by increased baPWV, even before the manifestation of structural arterial changes. Conversely, no significant difference in VFA was observed between the CIMT(+)baPWV(+) and CIMT(-)baPWV(+) groups, indicating that baPWV may be more sensitive than CIMT in detecting early functional vascular impairment, whereas CIMT is more related to structural abnormalities. These results are supported by a previous study involving 8, 839 Chinese patients with T2DM, demonstrating a significant correlation between VFA and baPWV (39). Similarly, Sun et al. also reported a positive correlation between VFA and arteriosclerosis (40). Although prior research has found that each 1% increase in HbA1c is associated with a 28% increased risk of macrovascular (41), our study did not observe similar results. This discrepancy may be attributed to differences in the glycemic control status of enrolled participants. The potential interaction between VFA and HbA1c in the progression of MVC warrants further investigation.

This study identified VFA as an independent predictor of MVC in patients with T2DM, corroborating findings from previous research (42, 43). Furthermore, a multicenter study reported that even among T2DM patients with normal BMI, those with VFA ≥90 cm² exhibited a significantly elevated 10-year risk of ASCVD, further reinforcing the central role of visceral adiposity in metabolic dysfunction (15).

Although VFA remained an independent predictor in our analysis in both male and female subgroups, notable sex-specific differences in associated confounding factors were observed. In female patients, HDL-C and LDL-C were significant predictors, whereas TG played a more prominent role in male patients. These differences may reflect the influence of sex hormone-mediated lipid metabolism and fat distribution patterns (44, 45), as well as variations in lifestyle and dietary habits (38).

The sex-specific predictive models developed in this study demonstrated strong discriminative ability, with an AUC of 0.905 for females (*P* < 0.001) and 0.870 for males (*P* < 0.001). These findings highlight the importance of incorporating sex-specific risk assessment strategies into clinical practice. Prior studies also support the existence of sex-based differences in arterial stiffness (39, 46). Notably, a large-scale cross-sectional study of the Chinese population reported a marked increase in baPWV levels in postmenopausal women (47).

Moreover, our study proposes a VFA cut-off value of 80.05cm² as a clinically relevant threshold for identifying individuals at elevated risk. This threshold is notably lower than the conventional criterion of VFA ≥100cm² for abdominal obesity (25), suggesting that early intervention should be considered once VFA reaches 80.05cm².

Obesity defined by BMI exhibits significant heterogeneity. Individuals with similar BMI values may present markedly different comorbidities and health risks (48, 49). With growing research into obesity-related complications, there has been increasing recognition of the critical role of visceral fat accumulation in disease progression (11–13, 33–38). VFA is considered the gold standard for diagnosing abdominal obesity. Although abdominal computed tomography (CT) and magnetic resonance imaging are commonly used for VFA assessment, the associated radiation exposure from CT and the high cost and time burden of magnetic resonance imaging limit their clinical application.

This study used dual BIA, a non-invasive, simple, and reproducible method for measuring VFA. Omura-Ohata et al. demonstrated that BIA could serve as an alternative to CT (50). Park et al. also found that the DualScan HDS-2000 device provided more accurate results in abdominal VFA compared to traditional whole-body BIA with CT (51). Our predictive model incorporated both BMI and VFA. The predictive efficacy of VFA for MVC was significantly greater than that of BMI (AUC: VFA = 0.74 vs. BMI = 0.612, *P<*0.001). This difference was especially pronounced in the female subgroup (AUC: VFA = 0.80 vs. BMI = 0.618, *P* < 0.001). Unlike the traditional obesity indicator BMI, which reflects overall body fat, VFA directly quantifies metabolically active visceral fat, the primary contributor to metabolic dysfunction. Consistent with previous studies (15, 39), our results support incorporating VFA measurement into routine diabetes complication risk assessment, particularly for individuals who are metabolically obese but with normal BMI. VFA assessment can help identify individuals with hidden cardiovascular risk.

Several limitations of this study should also be acknowledged. First, the cross-sectional design of this study limits the ability to infer causal relationships between VFA and arteriosclerosis. Longitudinal studies are required to investigate the dynamic changes in VFA and MVC over time. Second, the study sample was derived from a single center, which may limit the generalizability of the findings. Future multicenter studies are necessary to validate the robustness and applicability of the predictive model. Third, this study did not evaluate the potential influence of therapeutic interventions (e.g., pharmacologic treatment, lifestyle modifications) on macrovascular outcomes. Finally, given the retrospective nature of this study, there were differences in sample sizes among the three groups, which may have influenced the statistical power and interpretation of the comparisons, particularly in subgroup analyses. Although the methods used in the primary comparisons (such as logistic regression) are relatively robust to such imbalances, future studies should collect more balanced samples to verify the current findings.Nevertheless, all patients included in this cohort are part of an ongoing follow-up system, and prospective follow-up studies are planned to examine these factors in greater detail.

## Conclusions

VFA is an independent predictor of macrovascular complications in patients with T2DM, demonstrating superior predictive performance compared to BMI. The predictive model developed in this study—particularly the sex-specific models—showed high discriminative efficacy. These findings support the integration of VFA assessment into routine risk stratification for patients with T2DM. Implementing multidisciplinary approaches and refining risk classification strategies will be essential for the precise prevention and management of MVC in this population.

## Data Availability

The original contributions presented in the study are included in the article/**Supplementary Material**. Further inquiries can be directed to the corresponding author.
