# Supplementary material for: Visceral fat area as a predictor for macrovascular complications in patients with type 2 diabetes mellitus
Source: Front Endocrinol (Lausanne). 2026 Feb 12;17:1636998. doi: 10.3389/fendo.2026.1636998 (PMC12935640; doi:10.3389/fendo.2026.1636998)
Supplement: Supplementary file 1 [file SupplementaryFile1.docx]

| Variable | Step 1 OR(95%CI) | Step 2 OR（95%CI） | Step 3 OR（95%CI） | Step 4 OR（95%CI） | Step 5 OR（95%CI） | Step 6 OR（95%CI） | Step 7 OR（95%CI） | Step 8 OR（95%CI） | Step 9 OR（95%CI） | Step 10 OR（95%CI） | Step 11 OR（95%CI） | Step 12 OR（95%CI） | Step 13 OR（95%CI） | Final Model p-value |
| --- | --- | --- | --- | --- | --- | --- | --- | --- | --- | --- | --- | --- | --- | --- |
| Age | 1.077  (1.054-1.102) | 1.077  (1.054-1.101) | 1.077  (1.054-1.101) | 1.078  (1.055-1.101) | 1.079  (1.057-1.101) | 1.078  (1.057-1.1) | 1.079  (1.058-1.101) | 1.078  (1.057-1.099) | 1.077  (1.057-1.099) | 1.078  (1.057-1.099) | 1.077  (1.057-1.099) | 1.082  (1.061-1.102) | 1.086  (1.067-1.106） | <0.001 |
| SBP | 0.983  (0.961-1.005) | 0.983  (0.961-1.005) | 0.983  (0.961-1.005) | 0.983  (0.961-1.005) | 0.982  (0.961-1.004) | 0.983  (0.961-1.005) | 0.982  (0.961-1.004) | 0.983  (0.961-1.005) | 0.98  3(0.962-1.005) | 0.983  (0.962-1.005) | 0.983  (0.962-1.005) | - | - | - |
| DBP | 1.041  (1.027-1.056) | 1.041  (1.027-1.056) | 1.041  (1.027-1.056) | 1.041  (1.027-1.056) | 1.042  (1.027-1.056) | 1.041  (1.027-1.056) | 1.041  (1.027-1.056) | 1.041  (1.026-1.055) | 1.04  (1.026-1.055) | 1.041  (1.026-1.055) | 1.041  (1.027-1.055) | 1.033  (1.023-1.043) | 1.036  (1.026-1.045) | <0.001 |
| Weight | 0.975  (0.94-1.011) | 0.975  (0.94-1.011) | 0.975  (0.941-1.011) | 0.976  (0.941-1.011) | 0.976  (0.942-1.011) | 0.976  (0.942-1.011) | 0.975(0.941-1.01) | 0.966  (0.94-0.993) | 0.966  (0.94-0.993) | 0.968  (0.942-0.995) | 0.969  (0.943-0.995) | 0.969  (0.943-0.995) | 0.97  (0.945-0.997) | 0.028 |
| BMI | 0.889  (0.784-1.008) | 0.889  (0.784-1.008) | 0.889  (0.784-1.007) | 0.887  (0.783-1.005) | 0.887  (0.783-1.005) | 0.888  (0.784-1.006) | 0.889  (0.785-1.007) | 0.876  (0.778-0.987) | 0.878  (0.779-0.989) | 0.905  (0.817-1.003) | 0.895  (0.809-0.99) | 0.893  (0.808-0.988) | 0.897  (0.811-0.992) | 0.034 |
| Hip circumference | 0.969  (0.893-1.051) | 0.969  (0.893-1.051) | 0.968  (0.893-1.049) | 0.969  (0.894-1.05) | 0.967  (0.893-1.047) | 0.967  (0.893-1.047) | 0.969  (0.896-1.049) | - | - | - | - | - | - | - |
| W/H ratio | 280.267  (0.717-109559.344) | 282.344  (0.725-109993.117) | 293.516  (0.801-107544.35) | 283.619  (0.78-103129.846) | 308.909  (0.868-109953.073) | 342.309  (0.988-118550.789) | 250.393  (0.806-77772.422) | 39.309  (1.356-1139.315) | 39.119  (1.353-1131.227) | 42.484  (1.489-1211.817) | 43.218  (1.521-1227.758) | 41.099  (1.463-1154.403) | 41.119  (1.483-1139.991) | 0.028 |
| VFA | 1.064  (1.032-1.097) | 1.064  (1.032-1.097) | 1.063  (1.046-1.08) | 1.063  (1.046-1.08) | 1.063  (1.046-1.08) | 1.063  (1.046-1.08) | 1.063  (1.046-1.08) | 1.061  (1.045-1.077) | 1.061  (1.045-1.077) | 1.055  (1.044-1.067) | 1.055  (1.044-1.066) | 1.055  (1.044-1.066) | 1.055  (1.044-1.066) | <0.001 |
| SFA | 0.999  (0.985-1.014) | 0.999  (0.985-1.014) | - | - | - | - | - | - | - | - | - | - | - | - |
| V/S ratio | 0.228  (0.002-23.227) | 0.227  (0.002-23.15) | 0.274  (0.033-2.299) | 0.279  (0.033-2.33) | 0.274  (0.033-2.276) | 0.281  (0.034-2.323) | 0.277  (0.034-2.287) | 0.364  (0.05-2.67) | 0.36  4(0.05-2.667) | - | - | - | - | - |
| Course of disease | 1.004  (0.975-1.035) | 1.004  (0.975-1.035) | 1.004  (0.975-1.035) | 1.005  (0.975-1.035) | - | - | - | - | - | - | - | - | - | - |
| Hypertension | 0.714  (0.491-1.038) | 0.714  (0.491-1.038) | 0.714  (0.491-1.038) | 0.712  (0.49-1.035) | 0.708  (0.488-1.027) | 0.711  (0.491-1.03) | 0.711  (0.49-1.03) | 0.716  (0.494-1.036) | 0.705  (0.488-1.02) | 0.712  (0.493-1.03) | 0.727  (0.504-1.049) | 0.743  (0.516-1.07) | - | - |
| Hyperlipidemia | 0.861  (0.619-1.197) | 0.86  (0.619-1.197) | 0.861  (0.619-1.197) | 0.859  (0.618-1.194) | 0.86  (0.619-1.195) | 0.861  (0.619-1.196) | 0.862  (0.621-1.198) | 0.864  (0.622-1.2) | - | - | - | - | - | - |
| Fasting C-peptide | 1(0.999-1) | 1(0.999-1) | 1(0.999-1) | 1(0.999-1) | 1(0.999-1) | 1(0.999-1) | 1(0.999-1) | 1(0.999-1) | 1(0.999-1) | 1(0.999-1) | - | - | - | - |
| HbA1c | 1.017  (0.942-1.097) | 1.017  (0.943-1.096) | 1.016  (0.943-1.096) | 1.017  (0.943-1.096) | 1.014  (0.942-1.092) | - | - | - | - | - | - | - | - | - |
| γ-GT | 0.999  (0.995-1.002) | 0.999  (0.995-1.002) | 0.999  (0.995-1.002) | 0.999  (0.995-1.002) | 0.999  (0.995-1.002) | 0.999  (0.995-1.002) | - | - | - | - | - | - | - | - |
| BUN | 1.013  (0.908-1.131) | 1.014  (0.909-1.131) | 1.014  (0.909-1.131) | - | - | - | - | - | - | - | - | - | - | - |
| UA | 1  (0.998-1.002) | - | - | - | - | - | - | - | - | - | - | - | - | - |
| TG | 1.169  (1.033-1.323) | 1.17  (1.034-1.323) | 1.17  (1.035-1.323) | 1.171  (1.036-1.324) | 1.173  (1.038-1.326) | 1.176  (1.042-1.328) | 1.173  (1.039-1.324) | 1.174  (1.04-1.325) | 1.184  (1.05-1.334) | 1.181  (1.048-1.331) | 1.168  (1.038-1.313) | 1.161  (1.033-1.306) | 1.167  (1.038-1.312) | 0.01 |
